# How the Emotional Content of Discourse Affects Language Comprehension

**DOI:** 10.1371/journal.pone.0033718

**Published:** 2012-03-29

**Authors:** Laura Jiménez-Ortega, Manuel Martín-Loeches, Pilar Casado, Alejandra Sel, Sabela Fondevila, Pilar Herreros de Tejada, Annekathrin Schacht, Werner Sommer

**Affiliations:** 1 Center for Human Evolution and Behavior, UCM-ISCIII, Madrid, Spain; 2 Psychobiology Department, Complutense University of Madrid, Madrid, Spain; 3 Pyschobiology Department, Humboldt-University at Berlin, Berlin, Germany; 4 CRC Text Structures, University of Goettingen, Goettingen, Germany; University of Cambridge, United Kingdom

## Abstract

Emotion effects on cognition have often been reported. However, only few studies investigated emotional effects on subsequent language processing, and in most cases these effects were induced by non-linguistic stimuli such as films, faces, or pictures. Here, we investigated how a paragraph of positive, negative, or neutral emotional valence affects the processing of a subsequent emotionally neutral sentence, which contained either semantic, syntactic, or no violation, respectively, by means of event-related brain potentials (ERPs). Behavioral data revealed strong effects of emotion; error rates and reaction times increased significantly in sentences preceded by a positive paragraph relative to negative and neutral ones. In ERPs, the N400 to semantic violations was not affected by emotion. In the syntactic experiment, however, clear emotion effects were observed on ERPs. The left anterior negativity (LAN) to syntactic violations, which was not visible in the neutral condition, was present in the negative and positive conditions. This is interpreted as reflecting modulatory effects of prior emotions on syntactic processing, which is discussed in the light of three alternative or complementary explanations based on emotion-induced cognitive styles, working memory, and arousal models. The present effects of emotion on the LAN are especially remarkable considering that syntactic processing has often been regarded as encapsulated and autonomous.

## Introduction

For better or worse emotional states affect cognitive processing such as planning, attention, memory, creativity, problem solving, decision making, working memory, behavioral control, and language processing (e.g. [1], [2]). In everyday life, language is often used as a powerful tool to elicit emotions. Literature and poetry are magnificent examples of how language can make us feel deep emotions. Nevertheless, studies of emotional effects induced by language are still rare. Only recently, it has been shown that emotional processes elicited by language are in many aspects similar to those triggered by non-linguistic stimuli such as pictures, films, or faces [3], [4]. However, emotional effects elicited by language are traditionally thought to induce a lower amount of physiological arousal than non-linguistic stimuli [5], [6] and it also appears that both linguistic and non-linguistic emotional stimuli involve common, but also some different brain areas 3,7,8.The present study aims to investigate language-induced emotion effects on language comprehension, particularly semantic and syntactic processing.

Indeed, several lines of evidence support the possibility that language comprehension can be affected by emotional states. Overall, it has been shown that positive states increase the use of heuristic strategies, which are more global, category-level, and flexible. They are also computationally less demanding than algorithmic strategies, relying on pre-existing knowledge structures such as schemas and stereotypes, and not always taking into account all relevant information. In contrast, negative emotions promote the use of algorithmic strategies, which are more local, systematic, and more detail-oriented (e.g., [1], [9], [10], [11], [12], [13]). In line with this distinction, semantic processes involved in sentence comprehension have been characterized as heuristic, combining lexical items on the basis of plausibility, whereas syntactic processes appear to be more algorithmic, based on a finite list of well-defined instructions to combine lexical elements (reviewed in [14]). Consequently, modifying cognitive styles by means of induced positive or negative emotional states might differentially affect the way in which semantic and syntactic operations develop. In this sense, positive emotions might favor semantic processing, while negative emotions might favor syntactic processes. Detrimental effects of positive emotions on syntax and negative emotions on semantics could also be expected.

Alternatively, possible effects of emotion on language processes may also be predicted on the basis of models of working memory. In the literature, there are diverging views as to whether syntax and semantic information are stored in a language-specific or a general-purpose working memory system [15], [16], [17], and whether syntax and semantic information are stored in the same or in different working memory subsystems [15], [18], [19]. A constellation of possibilities appears therefore open. Overall, if a language-specific working memory system is held, emotions induced by language could affect linguistic processes more directly, or at least differently, as compared to emotions induced by other, non-linguistic means. Further, if the language-specific working memory system were partitioned into a system for syntactic information and a post-interpretive working memory system comprising (but not limited to) semantic information (e.g., [20]), differential effects of emotions could be expected on syntactic and semantic processes. A possibility in this regard is that syntactic processes would not be affected by emotions, as syntactic processing is often considered as encapsulated and largely unaffected by other cognitive operations [21], [22], [23], [24], [25], [26]. In turn, the more open nature of semantic processes would render them more susceptible for emotional states. The picture might nevertheless complicate a bit, since it has been proposed that although initial syntactic processes precede semantic processing, at later stages semantic and syntactic processes may interact, opening syntactic processes also to other sources of information [25], [27].

Finally, arousal induced by emotions might also be a source of effects on both semantic and syntactic processing. It has been reported that arousal induction is a crucial variable determining either facilitation or interference of linguistic tasks [28], [29], [30]. Ihssen et al. [29] observed that high arousing pictures were associated with an impaired processing of word targets in comparison to low arousing pictures regardless of picture valence. Furthermore, arousal activation modulates cognitive processes even at a very basic level such as conflict/error monitoring [31], [32]. However, previous investigations have failed to observe arousal effects on semantic processing, specifically in N400 and P600 components [33] . Accordingly, syntactic processing, which is considered a basic automatic process, could be more prone to be impaired than semantic processing as a consequence of the increasing arousal produced by emotional information.

A suitable way to study sentence comprehension processes is the recording of event-related brain potentials (ERPs). Their high temporal resolution allows real-time measurements of brain activity. In this regard, when semantic information is manipulated, a centro-parietal negativity called the N400 [34] appears approximately between 250 and 550 ms after the onset of a critical word. The N400 seems to reflect processes involved in the integration of word meaning into the sentence context, larger amplitudes being associated with increased integration difficulties [35]. If syntactic information is experimentally manipulated, left anterior negativities (LAN) have been described. The LAN to grammatical anomalies, such as morphosyntactic violations, appears between 300 and 500 ms, and apparently reflects highly automatic first parsing processes, the detection of a morphosyntactic mismatch, and/or the inability to assign the incoming word to the current phrase structure, as well as increased working memory demands related to syntactic structure [36], [37], [38], [39], [40]. Finally, a late component, a posterior positivity or P600, has been observed for both syntactic and semantic violations [41], [42], [43]. As this component is most robustly and consistently viewed after syntactic violations, it appears to reflect processing costs of repair and revision of structural mismatches [44], [45], but also integration processes between semantic and syntactic information [25], [46].

Effects of emotional states on language comprehension, using ERPs, have been studied mainly by observing effects of non-linguistic emotional stimuli. A number of these studies have explored the effects of emotional states on the N400 component. In most of them, the induced emotion and the emotional valence of the linguistic stimuli were opposite to each other, aiming to explore the effects of this type of incongruity on the N400 [47], [48], [49]. Federmeier et al. [11] using sentences pairs ending with the most expected word, an unexpected word from de expected semantic category, or an unexpected word from a different (related) category, investigated the effects of positive emotional states, induced by pictures, on the N400. For neutral conditions, they found smaller N400 amplitudes to unexpected words from an expected category than to unexpected words from a different category. However, under positive emotional conditions the N400 amplitude for these two types of unexpected words did not differ. The authors interpreted these results as the consequence of cognitive styles triggered by positive emotion, namely, facilitating semantic integration.

To the best of our knowledge, there is only one previous study, which investigated emotional effects on syntactic processing. Vissers et al. [13] reported that emotional states induced by means of emotional films affected the P600 to subject–verb agreement errors. Whereas positive emotions yielded a broader P600 distribution, negative emotions reduced the P600; no LAN was reported, and indeed emotional effects were absent in the LAN window. Vissers et al. [13] interpreted their results in the light of different processing styles induced by emotions, in combination with the complexity of the syntactic processing and attentional and/or motivational factors.

To sum up, previous investigations have reported that both semantic and syntactic processing are affected by emotions, although none of them used linguistic materials to induce emotions. As reasoned in this introduction, at least three theoretical frameworks – cognitive styles, working memory models, and arousal activity – might predict emotional effects on language processing, and some of them – particularly working memory - specific or differential effects when emotions are induced by language.

One main novelty of the present study is therefore the use of language to induce the emotions that may affect subsequent language processing. To this aim, we designed two different experiments. In the first one, we investigated the emotional effects produced by a positive, negative, or neutral paragraph on ERP components elicited by a semantic violation contained in a subsequent sentence of neutral valence. That is, we investigated to what extend the N400 and the P600 components would be affected by these manipulations. In the second experiment, we used the same paragraphs and neutral sentences, but included morphosyntactic rather than semantic violations, aiming at elucidating the effects of our procedures on the LAN and the P600 components.

Our experiments explore the effects of *emotions* on language, where emotional states are here generated by short paragraphs of different emotional valence, a procedure that has been seen to noticeably induce emotional states [3]. Positive and negative emotions may have differential effects on semantic and syntactic processes, predictions depending on the model approached. processes, depending on the point of view.

If a specific working memory system for language is assumed effects of emotion induced by language might be stronger than previously reported for emotions induced by non-linguistic stimuli. Further, if working memory for language is partitioned, emotions could affect syntax and semantics differentially. If the syntactic system is indeed encapsulated, one might expect that syntax processing is unaffected by emotion, particularly, at the earlier stages of syntactic processing as reflected by the LAN. According to the different cognitive styles scenario, different effects should be expected in the ERP components. In this regard, the N400 to semantic violations during positive emotional inductions might be reduced, indicating a facilitation of semantic integration during induced heuristic cognitive styles; in turn, a reduction of the LAN and P600 to syntactic violations could follow negative emotions. Finally, an increase of arousal level produced by the emotional paragraphs may impair both semantic and syntactic processing similarly, although differences in complexity between semantic and syntactic operations could also modulate the effects differentially, resulting in a stronger impairment of syntactic processing. Furthermore, no effects of arousal have been observed on N400 and P600 components [33]. Therefore, according to this framework, only the syntactic LAN component might be clearly affected.

## Experiment 1: Semantics

### Methods

#### Participants

Thirty native Spanish speakers (26 females, 4 males), aging from 18 to 35 years (mean 21.3), participated in the experiment. All of them had normal or corrected-to-normal vision and reported no history of reading difficulties or neural or psychiatric disorders. They were right-handed, with average handedness scores of 76%, ranging from 30 to 100%, according to the Edinburgh Handedness Inventory [54]. Participants were assessed with the Reading Span Test [50] scoring on average 3.6 and ranging between 1 and 6. The study was performed in accordance with the Declaration of Helsinki and approved by the ethics committee of the Center for Human Evolution and Behavior, UCM-ISCIII, Madrid, Spain. Prior to the experiment, participants gave their informed consent and were reimbursed thereafter.

#### Materials

We used 180 neutral sentences with the structure determiner–noun–adjective–verb, in which the adjectives were used as the critical words. Two versions of a given neutral sentence were constructed: a correct version and a noun-adjective semantic incompatibility (semantic violation). In addition, for each neutral sentence, we composed three preceding paragraphs of a related topic, with positive, negative, or neutral valence, respectively. The neutral sentences were always acceptable terminations for the corresponding paragraphs (Table S1). Each paragraph was composed by four simple short sentences containing on average 4.5, 4.3, and 4.2 words for positive, negative, and neutral paragraphs, respectively. Relative clauses and complex structures were avoided, favoring the use of frequent words, the most common structure being: subject, verb and complement. Accordingly, differences in difficulty between the three types of paragraphs are unlikely. Thirty Psychology graduate students, other than the sample of the main experiment, rated sentences and paragraphs for valence and arousal on a 5-point scale, ranging from 1 (negative or low arousing, respectively) to 5 (positive or high arousing, respectively). We used the following criteria for selecting the paragraphs: mean valence values greater than 3.8 were considered as positive and values below 2.2 as negative; mean valences between 2.5 and 3.5 were classified as neutral. The mean valence of selected (neutral) sentences was 2.9 (SD = 0.3) with mean arousal rating of 2.3 (SD = 0.3). Positive, negative, and neutral paragraphs obtained mean valence ratings of 4.1, 1.5, and 3.1 (SDs = 0.3, 0.3, and 0.2), respectively, and mean arousal ratings of 3.3, 3.4, and 2.4 (SDs = 0.6. 0.5, 0.4), respectively. Mean valence judgments for positive, negative, and neutral paragraphs were all significantly different (*t*s(179)>18, *p*s<.001). Arousal ratings for positive and negative paragraphs did not differ from each other (*t*(179) = .−17, *p = *.88) but were more arousing than neutral paragraphs (*ts*(179)>12, *ps*<.001).

A total of 1080 trials were created, 540 of which included a semantic violation in the neutral sentence. The remaining trials involved correct sentences. Trials were distributed in six different blocks. Each of them contained 90 correct and 90 incorrect sentences, which were preceded by negative, positive, or neutral paragraphs, evenly distributed. For a given participant, neither emotional paragraphs nor neutral sentences were repeated; paragraph-sentence combinations were presented in only one version (i.e., positive-correct, positive-incorrect, neutral-correct, neutral-incorrect, negative-correct, and negative-incorrect); the different versions were counterbalanced.

#### Procedure

Stimuli were presented white-on-black on an LCD screen, controlled by Presentation® Software. Participants' eyes were located 65 cm from the screen. Paragraphs were presented in the center of the screen, each sentence in a line; visual angles were around 3.5° in height and 8° to 14° in width. Neutral sentences were presented word-by-word in the center of the screen, with visual angles around 0.8° in height and 0.8° to 4° in width.

Participants performed a correctness judgment task on the neutral sentence by pressing one of two keys. Responses were given with the index and middle fingers of the left or right hands. Hand assignment to decision alternative was counterbalanced. In addition, after the key press a yes-no question about the paragraph content was presented in 20% of the trials, randomly distributed. This way, we ensured that participants attended to the paragraphs. They were also advised to avoid blinks during neutral sentence presentation in order to reduce ocular artifacts.

**Figure 1:**
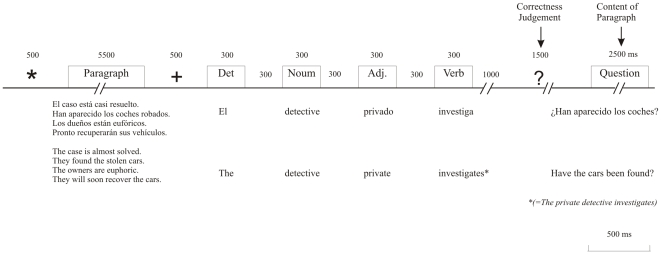
Schematic representation of the stimulation procedures. A positive, negative, or neutral paragraph was followed by a neutral sentence presented word by word, which was judged for correctness. Occasionally, a content question about the paragraph was presented thereafter to ensure attention to the paragraphs.

An asterisk signaled the beginning of a trial, which was followed by a paragraph presented in the center of the LCD for 5.5 s. Subsequently, a fixation cross appeared for 500 ms followed by the neutral sentence, which was presented word-by-word, with 300 ms exposition time per word and a stimulus onset asynchrony of 500 ms. After a 1 s interval, a question mark was presented for 1.5 s indicating the response period for the correctness judgment. Each neutral sentence was presented in the same form: the first word began with a capital letter and the last word was presented together with a period at the end. Questions about the paragraph were randomly presented during 2.5 s after the correctness response period, and the responses to these questions had to be verbalized. Thereafter, the inter-trial interval was 1 s (see Fig. 1).

A total of 180 sentences were presented in randomized order within a given block. Block presentation was counterbalanced, with each participant receiving only one of the six blocks. Each block consisted of 30 trials per condition (correct neutral trials, incorrect neutral trials, correct positive trials, incorrect positive trials, correct negative trials, incorrect negative trials) randomly presented. The session started with a training period including 24 trials other than those used in the experiment. Breaks were programmed after every 30 trials.

The electroencephalogram (EEG) was recorded from 27 tin electrodes embedded in an electrode cap (ElectroCap International) with a Brainamp amplifier. Electrode locations were: Fp1, Fp2, F7, F3, Fz, F4, F8, FC3, FC4, FT7, FT8, T7, C3, Cz, C4, T8, TP7, CP3, CP4, TP8, P7, P3, Pz, P4, P8, O1 and O2, plus right mastoid (M2), according to the extended 10/20 International System (American Electroencephalographic Society, 1991). All electrodes were referenced to the left mastoid (M1). Bipolar horizontal and vertical electrooculograms (EOG) were recorded for artifact monitoring. Electrode impedances were kept below 3 kΩ. The signal was continuously recorded with a bandpass from 0.01 to 30 Hz at a sampling rate of 250 Hz.

The data were re-referenced off-line to averaged mastoids. The continuous EEG record was divided into 1300 ms epochs, starting 200 ms before the onset of the adjectives within the neutral sentences and lasting 200 ms after the onset of the verbs. That is, epochs were time locked to the onset of the adjective, establishing a baseline starting at – 200 ms. Offline, artifacts were automatically rejected by eliminating epochs exceeding +/−100 µV in any of the channels. Blinks, vertical and horizontal eye movements were corrected using the method described by Gratton et al [51]. A visual inspection of the epochs was carried out in order to eliminate remaining epochs containing artifacts. Epochs enclosing incorrect responses (i.e., correct sentences judged as incorrect and vice versa) were also eliminated from data analysis. Overall, the mean rejection rate was 25.1% of the epochs and at least 22 trials could be analyzed for each condition.

ERPs were averaged separately for all conditions and electrodes. Mean amplitudes were measured in the following time windows: N400 (400 to 500 ms) and P600 (700 to 800 ms). Component windows were chosen according to the peaks in the difference waves.

Data were analyzed with repeated-measures ANOVAs and involved the factors Correctness (correct and incorrect sentences) and Emotional Content (positive, negative, and neutral preceding paragraphs). For the ERP-analyses, factor Electrode site was added, and the electrodes included in each analysis varied as a function of the region of interest for a given component. The region of interest for the N400 comprised C3, Cz, C4, P3, and P4 electrodes, whereas for the P600 they were C3, C4, P3, Pz, and P4. This way, we used equivalent numbers of electrodes to define regions of interest by covering the most representative regions for either component. Post-hoc tests were Bonferroni-corrected. Where appropriate, the Greenhouse-Geiser correction for violations of the sphericity assumption was applied.

### Results

#### Behavioral data

**Figure 2:**
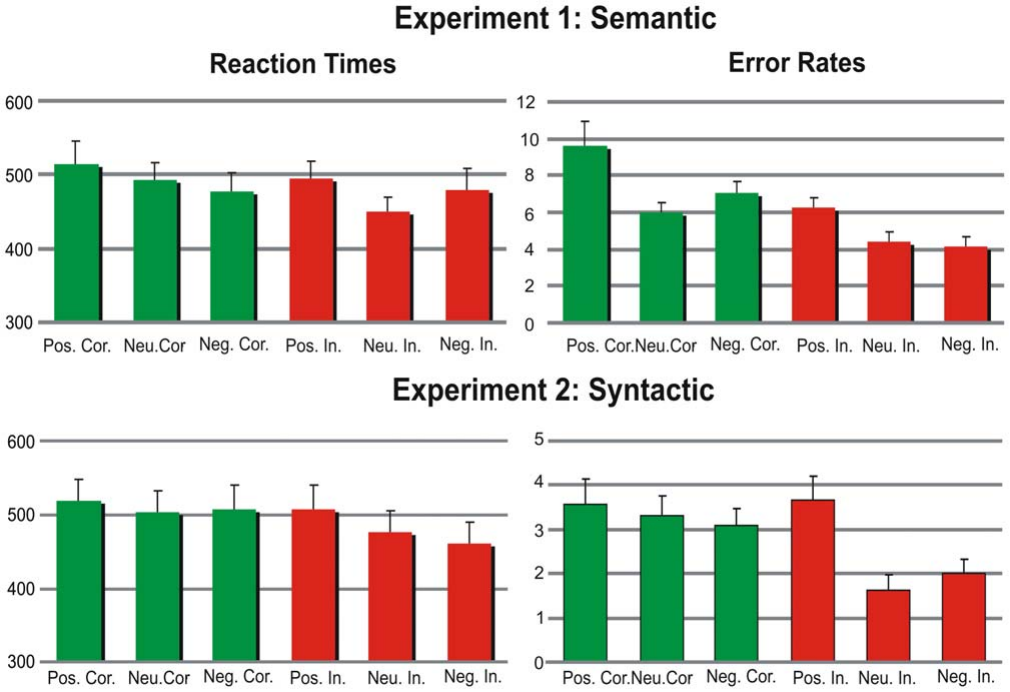
Semantic and syntactic error rates (experiment 1 and 2 respectively) and reaction times for positive negative and neutral correct (green) trials and for positive, negative and neutral incorrect trials (red).

Percentage of correct responses to the paragraph content question was high (89.5%) (Fig. 2). As regards sentence correctness judgments, Correctness effect was significant (*F*(1,29) = 11.3, *p*<.01), as mean error rates were larger for correct than for incorrect sentences (*M*s = 8.4 vs. 5.5%). A significant main effect of Emotion (*F*(2,58) = 14.29, *p*<.001) was found. Post-hoc analyses confirmed higher error rates for the positive condition as compared with negative and neutral conditions (*M*s = 13.2, 9.3, and 8.7%, respectively; *ps*<.01), whereas no differences were observed between neutral and negative conditions (*p>.1*). No significant Emotion by Correctness interaction was observed (*F*(2,58) = 2.06, *p* = .14).

There was also a main effect of Emotion on reaction times (*F*(2, 58) = 4.59, *p*<.01), which were longer in the positive than in both negative and neutral conditions (*Ms* = 505, 479, and 471 ms, respectively). Post-hoc tests confirmed significant differences between the positive and the neutral (*p*<.05), and a trend was observed between the positive and the negative condition (*p* = .08). No significant differences were observed between neutral and negative conditions (*p*>.1). Although average reaction times were somewhat longer for incorrect sentences in comparison with correct sentences (*M*s = 495 vs. 475 ms), no significant main effect of Correctness in reaction time was found (*F(*1,29) = 2.14, *p*>.1). No significant Emotion by Correctness interaction was observed (*F*(2,58) = .93, *p*>.1).

#### ERP data

**Figure 3:**
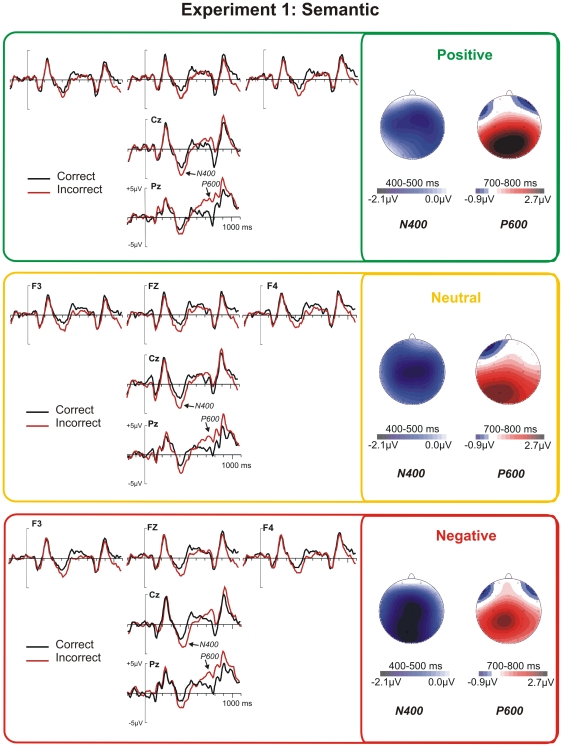
ERPs to semantically correct and incorrect adjectives under positive, neutral, and negative conditions. Left: ERP waveforms at selected electrodes. Right: Difference maps (incorrect minus correct) of the N400 and P600 in the analyzed time windows. Color scales of the maps are not symmetrical.

As can be seen in Figure 3, in the semantic experiment, the N400 peaked around 470 ms after adjective onset, and was maximal at central electrodes. This component was followed by a P600, maximal over parieto-central regions, starting at about 600 ms and peaking around 780 ms.

In the ANOVA on mean ERP amplitude between 400 and 500 ms, corresponding to the N400 component, we solely observed a significant main effect of Correctness (*F*(1,29) = 27.17, *p*<.001). The N400 therefore appeared to be unaffected by Emotion; neither a main effect of Emotion (*F*(2,58)<1), nor any interaction with Emotion was significant (all *F*s<1.2). Visual inspection of difference maps revealed the possibility of an enhanced negativity in O1 and O2 electrodes for incorrect words following negative paragraphs. To definitely discard any consistent emotional modulation of the N400, a separate ANOVA was performed on these two electrodes; but whereas a main effect of Correctness was observed (*F*(1,29) = 22.5, *p*<.001), there was again, no effect of Emotion, neither alone nor in interaction (all *Fs*<2.1).

Similar to the N400, the P600 was not affected by emotion. An ANOVA on mean ERP amplitudes between 700 and 800 ms showed significant effects of Correctness (*F*(1,29) = 24.98, *p*<.001), and an interaction between Correctness and Electrodes (*F*(4,116) = 8.49, *p*<.001). None of the other interactions was significant (all *F*s<1).

### Discussion

We investigated the influence of a positive, negative or neutral preceding paragraph on the processing of a subsequent neutral sentence containing a semantic violation. The participants' task was to decide whether the neutral sentence was correct or incorrect.

Behavioral data revealed clear emotional effects on performance. Error rates and reaction times were larger in the positive as compared to the neutral and negative conditions. This harmonizes well with previous reports that positive emotional states are associated with decrements in performance quality (e.g., [52]). It also indicates the success of our paragraphs in inducing emotions under the given experimental conditions. Error rates were also larger for correct than for incorrect sentences, which is a well-reported effect in the literature. (e.g., [42]). However, although error rates were higher for positive correct trials, we did not find a significant interaction between emotion and correctness, which matches with the lack of emotional effects observed in the ERP components subsequent to semantic violations.

As mentioned, no effects of emotions on the N400 component were observed here. This contrasts with the report by Federmeier et al. [11], who reported a significant N400 reduction under positive emotions in comparison to neutral states. We did not observe differences between positive and neutral conditions possibly due to methodological differences. In our study, we induced emotions by means of positive, negative, and neutral language, whereas Federmeier et al. [11], induced emotional states using positive and neutral pictures from the International Affective Picture System. Using those pictures, functional magnetic resonance studies revealed activation of middle temporal areas [7], [8], including BA 37 and 21, which also contribute to the N400 generation [53]. In contrast, emotions induced by short paragraphs mainly engage the nucleus accumbens, the medial prefrontal cortex, and the amygdala [3]. This can explain the different results between Federmeier et al. [11], and the present study, and further supports noticeable differences between emotional effects on cognitive processes as a function of the stimulus domain used to elicit emotions. The findings disparity between studies might be also explained by larger arousal levels observed in pictures in comparison to language [5], [6]. Nevertheless, as already mentioned, effects of arousal levels have not been observed either in N400 or in P600 components [11], [33].

In this regard, no effects on the P600 emerged here in the semantic condition. To our knowledge, emotional effects on the P600 elicited by semantic violations have not been previously reported, whereas the opposite holds for the same component to syntactic violations [13]. It is possible that the systematically smaller amplitude of this component to semantic violations when compared to syntactic violations, as has been the case here, prevents the emergence of observable modulations by emotions. This possibility can be better verified in our second experiment.

## Experiment 2: Syntactic

### Methods

#### Participants

Thirty native Spanish speakers (24 females and 6 males) with normal or corrected-to-normal vision participated in the experiment. Their mean age was 20.3 years (range 18 to 44). According to the Edinburgh Handedness Inventory [54], all were right handed (mean score: 70% ranging from 30% to 100%). In the Reading Span Test [50], a mean score of 3.7 (range 2 to 5) was obtained. The same ethical criteria as in the first experiment were fulfilled; participants gave their informed consent and were reimbursed.

#### Materials

Emotional paragraphs, neutral sentences and content questions were the same as in experiment 1 and they were also distributed in 6 blocks of 180 trials each (90 correct and 90 incorrect). However, for the incorrect trials, the neutral sentence contained a syntactic violation consisting in a noun-adjective gender disagreement.

#### Procedure and data analysis

The procedure was identical to the semantic experiment except for the data analysis. The overall mean rejection rate after artifact rejection and elimination of trials with incorrect responses was 21.3% of the epochs. The region of interest for the syntactic LAN component was F7, F3, Fz, F4, and F8 and the time window was 400–500 ms. The region of interest and time window for the P600 component was the same as in the semantic experiment (electrodes: C3, C4, P3, Pz and P4, time window: 700–800 ms).

### Results

#### Behavioral data

As in experiment 1, percentage of correct responses to the paragraph content question was high (95.5%) (Fig. 2). As regards sentence correctness judgments, ANOVA confirmed main effects of Emotion (*F*(1,29) = 6.32, *p*<.001), Correctness (*F*(2,58) = 9.64, *p*<.001), as well as of the Emotion by Correctness interaction (*F*(2,58) = 5.81, *p*<.01). In this regard, error rates were higher for the positive than for the negative and neutral conditions (*Ms* = 6.0, 4.0, and 3.5%, respectively). Error rates were also higher for correct than incorrect sentences (*M*s = 8.4 vs. 5.5%). Post-hoc analyses of the interactions revealed that whereas no significant differences were present between positive, negative, and neutral correct trials (*M*s = 3.5, 3.1, and 3.3, *p*s>.1), for incorrect trials error rates were higher in the positive than in the other two conditions (*M*s = 3.6, 1.4, and 1.6% for positive, negative and neutral, respectively; *p*s<.01), while the two latter conditions did not differ (*p*>.1).

There was no main effect of Emotion in reaction times (*F*(1,29) = 2.51, *p*>.05). However, as in the semantic experiment, Correctness was significant: reaction times were longer in correct sentences than in incorrect ones (*M*s = 511 vs. 482 ms; *F*(2,58) = 7.28, *p*<.01). The interaction between Correctness and Emotion was significant (*F*(1,29) = 7.25, *p*< = .01): whereas differences between correct sentences following positive, negative, and neutral paragraphs were not significant (*M*s = 508, 520, and 504 ms, *p*s>.1), within incorrect sentences, reaction times were significantly longer in the positive than in negative and neutral conditions (*M*s = 508, 462, and 478. ms, *p*s<.01), while they did not differ for negative and neutral conditions (*p*>.1).

#### ERP data

**Figure 4:**
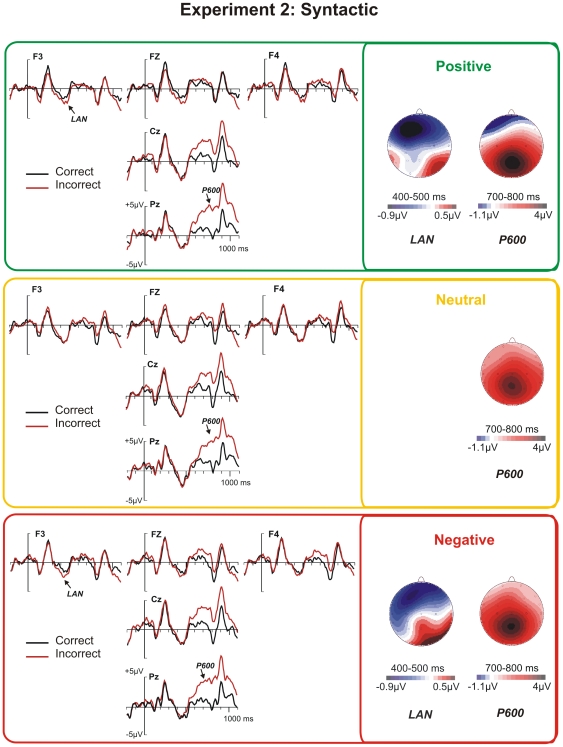
ERPs to syntactically correct and incorrect adjectives under positive, neutral, and negative conditions. Left: ERP waveforms at selected electrodes. Right: Differences maps (incorrect minus correct) of the LAN and P600 in the analyzed time windows. Color scales of the maps are not symmetrical.

In this experiment, a LAN component appeared, peaking between 400 and 500 ms, in the positive and negative conditions, but it was absent in the neutral condition (Figure 4). A repeated measures ANOVA at frontal electrodes (F7, F3, Fz, F4, and F8) revealed a significant Emotion by Correctness interaction (*F*(2,58) = 3.9, *p*<.05), whereas no significant main effects were observed neither for Emotion or Correctness as main effects, nor in interaction with Electrode (all *F*s<1.2). Post-hoc analyses showed significant differences between the positive and neutral condition (*F*(1,29) = 6.22, *p*<.05), and a fairly strong trend between the negative and neutral condition (*F*(1,29) = 3.87, *p* = .059), whereas no difference between the negative and positive conditions was observed (F(1,29) = .94, p>0.5).

With the aim of maximizing statistical power and in order to confirm the observed trend, post-hoc statistical analyses were performed using the electrodes where the LAN was maximal, F3 and Fz. A first analysis explored Correctness effects as a function of the emotional condition separately, which yielded significant effects of Correctness in the positive and negative conditions (*F*(1,29) = 5.7, *p*<.01, and *F*(1,29) = 2.2, *p*<.05, respectively), but not in the neutral condition (*F*(1,29)<1). Further, the LANs in the positive and negative conditions were compared by means of a post-hoc ANOVA which revealed no effects of Emotion, neither alone nor in interaction (all Fs<1), whereas Correctness was significant (*F*(1,29) = 6.5, *p*<.05). Accordingly, the LAN was apparent in the two emotional conditions and did not differ between positive and negative emotions.

In the 700–800 ms window, a large P600 component appeared, which was confirmed by a significant main effect of Correctness (*F*(1,29) = 24.98, *p*<.001) and its interaction with Electrode (*F*(26,754) = 8.49, *p*<.001). However, effects of Emotion, whether alone or in interactions, were absent (all *F*s<1).

### Discussion

We investigated the influence of a positive, negative or neutral preceding paragraph on the processing of a subsequent neutral sentence containing a syntactic violation. The participants' task was to decide whether the neutral sentence was correct or incorrect.

As in the semantic experiment, behavioral data yielded overall higher error rates for the positive condition. Additionally, error rates and reaction times were larger in positive incorrect sentences in comparison with neutral and negative ones; that is, subjects exhibited problems to detect errors in the positive condition. Effects of emotions on correct sentences did not emerge, however. This indicates not only that our paragraphs efficiently elicit emotional effects, but that these effects interacted with the linguistic task in this (syntactic) experiment.

Emotional effects on the LAN seem to be quite remarkable. First, the LAN was not observed in the neutral condition. This is not an uncommon result; numerous studies have failed to observe anterior negativities to morpho-syntactic anomalies (e.g., [55], [56], [57], [58], [59], [60], [61], [62]). Indeed, some authors have claimed that the LAN is so small that it may be prone to statistical power problems [62]; but it is also the case that in a number of studies it could not be seen at all (e.g., [13]). Accordingly, we do not assume that there was no LAN at all after neutral paragraphs in the present experiment; however, the LAN was boosted after emotional paragraphs, suggestive of a straight modulation by emotions of the first-pass syntactic processes presumably reflected by this component.

Emotions, generated by short text paragraphs, may increase the difficulty of early syntactic processing resulting in the LAN modulation. In this line, they have been interpreted previous boosting effects on language-related ERP components [37], the opposite being true for amplitude reductions [11]. Our behavioral data are in consonance with this interpretation, as syntactic error detection appeared to be harder in the presence of positive emotions. However, although the LAN indicated similar difficulties under the negative condition, behavioral data did not align with this suggestion. This might indicate that the initial difficulties may be overcome during the period between initial syntactic processing and the response. Behavioral effects observed here might therefore be better linked to later processes related to participants' responses.

Traditionally, syntactic processing has been regarded as an automatic and encapsulated operation (e.g., [22], [23], [24]. According to Hagoort [37], semantics is affected by syntax but not vice versa, in author's words: “Syntax is selfish, whereas semantic is altruistic” (p. 897). Moreover, Friederici et al. [25] proposed that initial syntactic structure precedes and is in many respects independent of semantic processing. Even though, evidence that other types of information, such as semantics or context, can affect early syntactic processing has been obtained (e.g., [42], [63]). In line with this, our experimental results demonstrated emotional effects on early syntactic processing. Thus, syntax processing might interact with other domains even at early stages, and might therefore be less independent and encapsulated than traditionally postulated.

As in the semantic experiment, emotional effects were not observed on the P600, even though this component displayed its maximal amplitude in this experiment. This contrasts with the strong reduction of the P600 to subject–verb agreement violations under sad emotions in comparison to positive emotional conditions in Vissers et al. study [13]. In the latter study, however, emotions were induced by happy or sad film clips, whereas we used short paragraphs of text. The use of non-linguistic vs. linguistic stimuli to induce emotions and their impacts on language processing might account for the different results. These domain-specific effects might not only explain the absence of P600 modulations by emotion in the present study but could also be the reason for the absence of LAN modulations in the study of Vissers et al. [13]. This will be reconsidered further in the following general discussion.

## Discussion

The present study aimed to investigate the effect of emotion, induced by short paragraphs of text, on semantic and syntactic processes in sentence comprehension. To this objective, emotionally neutral sentences were presented directly after the text paragraphs and contained either semantic (Experiment 1) or syntactic (Experiment 2) violations.

Overall, short paragraphs can successfully elicit emotional states that impact sentence processing. Positive emotions impaired both syntactic and semantic error detection task in comparison to negative and neutral emotions. We have also found that emotions elicited by short paragraphs did not affect specifically semantic processes occurring during the course of sentence processing, as reflected in the N400 ERP component. However, the same text paragraphs were able to noticeably modulate the LAN component, presumably reflecting early syntactic processes. In contrast, the P600 component, probably an index of revision or repair of an anomalous sentence structure, in which both syntactic and semantic information might be integrated, was not affected by our manipulations. Accordingly, emotions induced by text seem to affect first-pass parsing operations, and this apparently in a similar manner irrespective of the valence (positive, negative) of the emotion elicited.

We will discuss our results in the light of the three different - though not mutually exclusive - scenarios outlined in the introduction, viz., emotion-induced cognitive stiles, working memory models, and arousal levels.

Through the discussion we will address the results of both semantic and syntactic conditions jointly, given that subject samples were very similar. In this regard, all of them were university students of comparable age and participants in both groups showed, on average, similar performance in the Reading Span Test.

As mentioned in the introduction, positive emotional states seem to increase the use of global, heuristic, and flexible strategies, while negative affects increase the use of algorithmic strategies (e.g., [1], [9]). In line with this, we predicted an N400 reduction to semantic violations following positive paragraphs, reflecting facilitating effects on semantic integration by inducing more heuristic cognitive styles. In the same line of reasoning, the LAN in the second experiment as well as the P600 in both the first and the second experiments might be reduced under negative emotions. None of these results was found, however. In the present study, regarding the ERP components reflecting linguistic operations, the overall similarity between positive and negative emotional states suggests that changes in cognitive styles cannot account for the present results. On the other hand, behavioral data showed higher error rates during positive conditions in both the semantic and the syntactic experiment, and only in the syntactic one this interacted with sentence correctness. As argued in the corresponding discussions of Experiments 1 and 2, these findings appear to be related to more general detrimental effects of positive emotional states on performance [64] and factors unfolding during the response period than to factors developing along core language processes. In fact, behavioral data are better explained by the cognitive styles account than ERP data.

It can be argued that our short paragraphs induced emotional states not enduring enough as to produce obvious changes in cognitive styles, as these are traditionally considered long-lasting responses. Indeed, a block-wise presentation may be more appropriate to induce moods or long-term emotional states than the present procedure, in which emotional valence of the paragraphs varied from trial to trial. However, in our opinion, at least some processes elicited by emotional states have been triggered in the present study, as revealed by the behavioral responses of the participants. It might be the case, therefore, that emotional states affect other, non-linguistic processes of the task while leaving core linguistic processes unaffected. Further research is needed to clarify this possibility.

Our results also convey specific implications when approaching the question from working memory accounts. The fact that only early syntactic processes but neither later syntactic nor semantic processes appeared to be affected appears compatible with a partitioning of the working memory system into several relatively independent subsystems (reviewed in the introduction). In this regard, even subsystems devoted to syntax processing might be partitioned into early and late stages, reflected in differential effects for the LAN and the P600 components. As discussed in the context of Experiment 2, our data also add to recent evidence (e.g., [42]) indicating that syntax, specifically earlier operations within this domain, as reflected in the LAN component, may not be as encapsulated and opaque to other sources of information as traditionally assumed (e.g., [22], [23], [24], [25]).

When considering previous reports (e.g., [11], [13]), our results are also compatible with a distinction between working memory systems for language as separate and relatively independent from other working memory systems. At variance with our results, Vissers et al. [13] did not show LAN modulations by emotional states, but reported modulations of the P600 instead. Two important differences between Vissers et al. [13] and the present study might actually account for the apparent contradictory results. One is the use of non-linguistic stimuli (video clips) to elicit emotional states by Visser and colleagues, whereas we have used linguistic material instead. As already mentioned, these domain-specific effects might not only explain the absence of P600 modulations by emotion in the present study but could also be the reason for the absence of LAN modulations in the study of Vissers et al. [13]. Indeed, as stated in the introduction, linguistic and non-linguistic emotional stimuli involve common but also different brain areas [3], [7], [8], this possibly accounting for differential effects as a function of the domain used to elicit emotional states. On the other hand, Vissers et al. [13] induced emotional states of relatively long duration - elicited by expositions of materials lasting nearly 6 minutes -, contrasting with the emotional paragraphs of brief duration in our study. Exactly the same rationale would apply to the differences between the present study and the one by Federmeier et al. [11], in which the N400 component appeared reduced after inducing positive moods with pictures. Accordingly, in Vissers et al. [13] and Federmeier et al. [11] studies mood modulations may have been effectively manipulated, whereas –as discussed above- this is not so apparent in the present study. Differences in the combination of domain-specific and durability of the emotion eliciting material could explain some of the differences between the present and previous studies.

In our view, the arousal activity account applies very accurately and parsimoniously to our ERP results. As detailed in the introduction, arousal levels might lead either to facilitation or impairment of cognitive tasks [28], [29], [30], [31], [32]. Further, previous investigations have failed to demonstrate arousal effects on N400 or P600 components [33]. Therefore, high arousal levels induced by positive and negative paragraphs might differentially affect semantic and syntactic processing. In fact, we observed effects exclusively on the LAN component, and they did not differ between the emotional valences of the paragraphs. Most probably, detecting syntactic errors is a more automatic task generating fewer conflicts than detecting semantic errors. Therefore, the boost of the LAN component for the positive and negative conditions is parsimoniously explained by arousal activation produced by the preceding emotional information contained in the paragraph. Accordingly, whereas behavioral data seem to be better explained by a cognitive styles account, ERP data reflecting core linguistic processes appear better explained by the arousal activity account. This would be indication that both phenomena, i.e., alterations of arousal level and cognitive style, appeared to coexist in our data and that both seemed to exert differential effects, harmonizing well with the fact that whereas ERPs (namely, the LAN) reflect only one aspect of information processing, performance is actually the end result of a number of subprocesses, including response decisions.

In conclusion, emotional states can be induced by short paragraphs of emotional content and seem to reliably modulate language comprehension. This is so even if the emotional valence of the paragraphs changed continuously on a trial-to-trial basis. Modulations of cognitive processes have been robust enough as to affect participants' responses both at the neurophysiological (ERPs) as well as the behavioral level. A relatively complex set of modulations, at least comprising changes in arousal levels and cognitive styles, may coexist, as the effects on ERP fluctuations reflecting specific linguistic operations and performance results were differentially affected. These effects provide valuable evidence to be taken into account to better understand the multiple processes involved in countless daily situations, including reading, conversations, or speech acts.

## Supporting Information

Table S1 Examples of preceding positive, negative, and neutral paragraphs and the subsequent neutral sentences containing either semantic or syntactic violations used for both the first and the second experiments, respectively.
(DOC)
