# Supplementary material for: How the Emotional Content of Discourse Affects Language Comprehension
Source: PLoS One. 2012 Mar 29;7(3):e33718. doi: 10.1371/journal.pone.0033718 (PMC3315581; doi:10.1371/journal.pone.0033718)
Supplement: Table S1 — Examples of preceding positive, negative, and neutral paragraphs and the subsequent neutral sentences containing either semantic or syntactic violations used for both the first and the second experiments, respectively. (DOC) [file pone.0033718.s001.doc]

|  | *Task 1* | | | | | *Task 2 (20% of the trials)* |
| --- | --- | --- | --- | --- | --- | --- |
| *Condition* | *Paragraph* | *Det.* | *Noun* | *Adj.* | *Verb* | *Content Question* |
| *Positive* |  |  |  |  |  |  |
| *Correct* | El caso está casi resuelto. |  |  | privado |  |  |
| *Semantic* | Han aparecido los coches robados. | El | detective | privados | investiga | ¿Han aparecido los coches? |
| *Syntactic* | Los dueños están eufóricos. |  |  | acuoso |  |  |
|  | Pronto recuperarán sus vehículos |  |  |  |  |  |
| *Neutral* |  |  |  |  |  |  |
| *Correct* | Aparca el coche. |  |  | privado |  |  |
| *Semantic* | Enciende la radio. | El | detective | privados | investiga | ¿Ha encendido la radio? |
| *Syntactic* | Espera pacientemente. |  |  | acuoso |  |  |
|  | Observa atentamente la casa |  |  |  |  |
| *Negative* |  |  |  |  |  |  |
| *Correct* | El crimen tuvo lugar en esta calle. |  |  | privado |  |  |
| *Semantic* | El chaval fue acorralado. | El | detective | privados | investiga | ¿Le pegaron una paliza? |
| *Syntactic* | Le pegaron una paliza. |  |  | acuoso |  |  |
|  | Después fue acuchillado. |  |  |  |  |  |
|  |  |  |  |  |  |  |
|  |  |  |  |  |  |  |
| *Positive* |  |  |  |  |  |  |
| *Correct* | The case is almost solved. |  |  | private_[sing.]_ |  |  |
| *Semantic* | They found the stolen cars. | The | detective | private _[pl.]_ | investigates* | ¿Have the cars been found? |
| *Syntactic* | The owners are euphoric. |  |  | watery |  |  |
|  | They will soon recover the cars |  |  |  |  |  |
| *Neutral* |  |  |  |  |  |  |
| *Correct* | He parks the car. |  |  | private_[sing.]_ |  |  |
| *Semantic* | He turns on the radio. | The | detective | private _[pl.]_ | investigates* | ¿Has he turned on the radio? |
| *Syntactic* | He waits patiently. |  |  | watery |  |  |
|  | He looks out at the house |  |  |  |  |  |
| *Negative* |  |  |  |  |  |  |
| *Correct* | The crime took place in this street |  |  | private_[sing.]_ |  |  |
| *Semantic* | The youngster was cornered | The | detective | private _[pl.]_ | investigates* | ¿Did they bit him? |
| *Syntactic* | They bit him up |  |  | watery |  |  |
|  | Them, he was stabbed |  |  |  |  |  |
|  |  |  |  |  |  |  |
| **(=The private*_[sing.]_*/* *private* _[pl.]_/*watery detective investigates)*  *Where sing.= singular and pl.= plural* | | | | | | |
